# Vascular Risk Status as a Predictor of Later-Life Depressive Symptoms: A Cohort Study

**DOI:** 10.1016/j.biopsych.2012.02.005

**Published:** 2012-08-15

**Authors:** Mika Kivimäki, Martin J. Shipley, Charlotte L. Allan, Claire E. Sexton, Markus Jokela, Marianna Virtanen, Henning Tiemeier, Klaus P. Ebmeier, Archana Singh-Manoux

**Affiliations:** a Department of Epidemiology and Public Health, University College London, London, United Kingdom; b Department of Psychiatry, University of Oxford, Warneford Hospital, Oxford, United Kingdom; c Finnish Institute of Occupational Health and University of Helsinki, Helsinki, Finland; d Departments of Epidemiology and Psychiatry, Erasmus Medical Center, Rotterdam, the Netherlands; e Institut National de la Santé et de la Recherche Médicale, Paris, France

**Keywords:** Aging, cardiovascular risk factors, depressive symptoms, late-onset depression, risk prediction, vascular depression

## Abstract

**Background:**

Common etiology of vascular diseases and later-life depression may provide important synergies for prevention. We examined whether standard clinical risk profiles developed for vascular diseases also predict depressive symptoms in older adults.

**Methods:**

Data were drawn from the Whitehall II study with baseline examination in 1991; follow-up screenings in 1997, 2003, and 2008; and additional disease ascertainment from hospital data and registry linkage on 5318 participants (mean age 54.8 years, 31% women) without depressive symptoms at baseline. Vascular risk was assessed with the Framingham Cardiovascular, Coronary Heart Disease, and Stroke Risk Scores. New depressive symptoms at each follow-up screening were identified by General Health Questionnaire caseness, a Center for Epidemiologic Studies Depression Scale score ≥16, and use of antidepressant medication.

**Results:**

Diagnosed vascular disease (that is, coronary heart disease or stroke) was associated with an increased risk for depressive symptoms, age- and sex-adjusted odds ratios from 1.5 (95% confidence interval 1.0–2.2) to 2.0 (1.4–3.0), depending on the indicator of depressive symptoms. Among participants without manifest vascular disease, the Stroke Risk Score was associated with Center for Epidemiologic Studies Depression Scale depressive symptoms before age 65 (age- and sex-adjusted odds ratio per 10% absolute change in the score = 3.1 [1.5–6.5]), but none of the risk scores predicted new-onset depressive symptoms in those aged ≥65 (odds ratios from .8 to 1.2).

**Conclusions:**

These data suggest that public health measures to improve vascular risk status will influence the incidence of later-life depressive symptoms via reduced rates of manifest vascular disease.

The vascular (or subcortical ischemic) depression hypothesis suggests a link between vascular pathology and later life depression (1–4). The mechanisms underlying this link are thought to involve microdamage in small vessels in the frontal and subcortical regions of the brain, which are related to mood regulation, and also hypothesized to contribute to the development of depressive states (5). Epidemiological evidence shows vascular diseases, such as myocardial infarction and stroke, increase the risk of depression (6). Recently, Mast *et al.* (7) also found a composite score combining vascular diseases and vascular risk factors to predict 2-year incidence of elevated depressive symptoms in individuals aged 70 to 79, a finding replicated in another cohort of elders (8). However, few studies have been based on long follow-ups (1,2,9–11), and it is not known whether vascular risk factors in the absence of manifest vascular disease predict later-life depression risk among middle-aged individuals. The clinical significance of establishing a link between vascular risk status and later-life depression is in relation to prevention of mental disorders.

Current guidelines on risk management and treatment of vascular diseases recommend use of multifactorial risk prediction algorithms (12–14), the Framingham risk scores being the most widely used and validated in clinical settings (15–17). These scores incorporate data on routinely measured risk factors, such as lipid levels, blood pressure, and smoking, to calculate risk of vascular disease at the individual level. The Framingham risk scores have been shown to predict incident coronary heart disease (CHD), stroke, and cognitive decline (15–18), but whether they also predict onset of depressive symptoms at older ages is unknown.

In this study, we first examine the status of diagnosed vascular disease as a risk factor for depressive symptoms. We hypothesize that this association exists; this is in agreement with the current clinical guidelines that recommend screening patients with vascular disease for depressive symptoms (19–21). The second objective of this study is to examine whether in the absence of manifest cardiovascular disease (CVD), subclinical vascular risk status, measured with standard clinical risk scores developed for assessing risk of vascular disease, predicts depressive symptoms in older adults. An increased risk of depressive symptoms among persons with an adverse preclinical cardiovascular profile would suggest that public health measures to improve vascular risk status will also reduce prevalence of depressive symptoms.

## Methods and Materials

### Study Population

The Whitehall II study is a prospective cohort study of British civil servants established in 1985 to study associations between risk factors, pathophysiological changes, and clinical disease (22). The target population was all London-based office staff, aged 35 to 55, working in 20 civil service departments on recruitment to the study in 1985 to 1988 (phase 1). With a response of 73%, the cohort consisted of 10,308 employees (6895 men and 3413 women). Since the phase 1 medical examination, follow-up examinations have taken place approximately every 5 years: phase 3 (1991 to 1993, *n* = 8815); phase 5 (1997 to 1999, *n* = 7870); phase 7 (2003 to 2004, *n* = 6967); and phase 9 (2008 to 2009, *n* = 6761). Phase 3 was the first time that all components of the Framingham risk algorithms were measured, making it the baseline for the analyses we report here. We use data from three screening cycles: from phase 3 to phase 5, from phase 5 to phase 7, and from phase 7 to phase 9 with phases 3, 5, and 7 providing baseline measures of vascular risk to assess incidence of depressive symptoms at phases 5, 7, and 9 in the three data cycles, respectively.

Ethical approval for the Whitehall II study was obtained from the University College London Medical School committee on the ethics of human research; all participants provided written informed consent.

### Ascertainment of Coronary Heart Disease, Stroke, and Nonvascular Chronic Conditions

Prevalent CHD (a history of myocardial infarction or angina), stroke, and nonvascular chronic conditions were ascertained at phases 3, 5, and 7. A history of angina was identified via questionnaire and was corroborated with medical records, by abnormalities in a resting electrocardiogram (ECG), an exercise ECG, or a coronary angiogram. Nonfatal myocardial infarction was defined following the World Health Organization Multinational Monitoring of Trends and Determinants in Cardiovascular Disease criteria (23) and ascertained using data from five yearly medical examinations, hospital records of acute ECGs, and use of cardiac enzymes. A history of stroke was ascertained by self-reports (“Have you ever been told by a doctor that you have had a stroke or transient ischemic attack?” Yes/No). Nonvascular chronic condition in participants free of CHD and stroke was identified by an affirmative response to a question: “Do you have any longstanding illness, disability or infirmity?”

### Assessment of Framingham Risk Factors

We used standard operating protocols to measure risk factors for the Framingham general CVD risk score (sex, age, diabetes, smoking, treated and untreated systolic blood pressure, total cholesterol, high-density lipoprotein [HDL] cholesterol), CHD risk score (sex, age, diabetes, smoking, systolic and diastolic blood pressure, total cholesterol, HDL cholesterol), and stroke risk score (sex, age, systolic blood pressure, diabetes, smoking, prior cardiovascular disease, atrial fibrillation, left-ventricular hypertrophy, use of hypertensive medications) at the baseline of each data cycle, i.e., phases 3, 5, and 7 (14–17). Venous blood was taken in the fasting state or at least 5 hours after a light, fat-free breakfast. Serum for lipid analyses was refrigerated at −4°C and assayed within 72 hours. Cholesterol was measured with the use of a Cobas Fara centrifugal analyzer (Roche Diagnostics System, Nutley, New Jersey). High-density lipoprotein cholesterol was measured by precipitating non-HDL cholesterol with dextran sulfate-magnesium chloride using a centrifuge and measuring cholesterol in the supernatant. Participants underwent an oral glucose tolerance test and new venous blood samples were taken at 2 hours postadministration of a 75 g glucose solution. Blood glucose was measured using the glucose oxidase method on a YSI MODEL 2300 STAT PLUS Analyzer (YSI Corporation, Yellow Springs, Ohio; mean coefficient of variation: 1.4%–3.1%). Diabetes was defined by fasting glucose ≥7.0 mmol/L or 2-hour postload glucose ≥11.1 mmol/L, reported doctor diagnosed diabetes, or use of diabetes medication (24). We measured systolic and diastolic blood pressure twice in the sitting position after 5 minutes rest with a Hawksley random-zero sphygmomanometer (phases 3 and 5) (Lynjay Services Ltd., Worthing, United Kingdom) and OMRON HEM 907 (phase 7) (Omron, Milton Keynes, United Kingdom), the average of two readings used in the analysis. Atrial fibrillation was identified on the Glasgow 12-lead electrocardiogram analysis program combined with manual review (Prof. P. Macfarlane, University of Glasgow, United Kingdom). Limb lead electrocardiograms were classified according to the Minnesota code (25), with tall R waves (codes 3-1 to 3-3) used to reflect left ventricular hypertrophy. Information on smoking and use of antihypertensive drugs and lipid-lowering medication was requested at phases 3, 5, and 7.

The validity of the Framingham risk scores as measures of vascular risk was supported in our study, as they strongly predicted incidence of subsequent (manifest) vascular disease. Age- and sex-adjusted odds ratio for 10% increment in risk was 2.84 (95% confidence interval [CI] 1.66–4.88) for the stroke risk score-incident stroke association and 2.25 (95% CI 1.92–2.65) for the CHD risk score-incident CHD association. The corresponding odds ratios for the associations of CVD risk score with stroke and CHD were 1.34 (95% CI 1.11–1.62) and 2.14 (95% CI 1.85–2.48), respectively.

### Assessment of Depressive Symptoms

We used three indicators (or proxy measures) to identify persons with depressive symptoms. First, participants responded to the self-administered 30-item General Health Questionnaire (GHQ) at phases 3, 5, 7, and 9, a screening instrument designed for and widely used in population-based surveys and trials (26). Each questionnaire item enquires about a specific symptom, with response categories scored as either 1 or 0 to indicate whether the symptom is present. Total score of 5 or more led to individuals being defined as GHQ-symptom cases and scores 0 to 4 as noncases (27). Although the GHQ was originally designed to assess depressive and anxiety symptoms, a recent population-based study showed GHQ caseness to be sensitive (84%) and specific (84%) in detecting dysthymia or major depressive disorder, as indicated by the Composite International Diagnostic Interview (28). The GHQ has also been validated at baseline against a clinical interview schedule in the Whitehall II study, with acceptable sensitivity (73%) and specificity (78%) (27). In a more recent validation using a subgroup of 274 participants aged 58 to 70 in 2010, the sensitivity and specificity of GHQ symptom caseness against diagnosed depressive episodes based on a structured psychiatric interview were 80% and 81%, respectively.

Second, at phases 7 and 9, depressive symptoms were also assessed using the Center for Epidemiologic Studies Depression Scale (CES-D) (note that CES-D was not included in earlier screening phases of our study) (29). The 20 items of the CES-D measure symptoms associated with depression; participants are asked to score the frequency of occurrence of specific symptoms during the previous week on a four point scale (0 = less than one day, 1 = 1–2 days, 2 = 3–4 days, and 3 = 5–7 days). These items are summed to yield a total score between 0 and 60, with participants scoring ≥16 defined as having CES-D depressive symptoms (30). In a validation study of 274 Whitehall II participants, sensitivity and specificity were 89% and 86%, respectively, for CES-D depressive symptoms using a structured psychiatric interview as the criterion.

Third, at phases 3, 5, 7, and 9, participants were asked whether they had taken any medication in the past 14 days and, if so, to provide the name of the medication. Medications were coded using British National Formulary codes to define antidepressant medication use (codes: 040301–040304) (31).

### Statistical Analysis

All data analyses were performed with SAS version 9.2 (SAS Institute Inc., Cary, North Carolina). As the two study questions addressed in this investigation require different population samples, we describe the analytic samples and procedures in two parts. The analyses combine men and women, as there was no evidence to suggest that the associations of vascular disease and the Framingham risk scores with subsequent depressive symptoms differed by sex (all *p* values for sex interaction > .07). The analysis strategy for repeated data cycles followed that used in the Framingham study (32).

#### Analysis 1: Manifest Vascular and Nonvascular Disease as a Predictor of Depressive Symptoms

Participants were eligible for these analyses if they completed the health questionnaire at any two consecutive phases between phase 3 and phase 9 and had no missing data on disease status at the corresponding baseline phases (see Supplement 1 for a detailed description of sample selection). For comparison, we examined the association between nonvascular diseases as a predictor of depressive symptoms. Briefly, at each of the three data cycles, we excluded participants who had prevalent or previous depressive symptoms at baseline or missing data on depressive symptoms at baseline or at follow-up. Each participant could contribute to the outcome (onset of depressive symptoms) only once. The analysis of GHQ symptoms was based on 5230, 4029, and 3571 participants in the first, second, and third data cycles, a total of 12,830 person observations. The corresponding numbers for the analysis of antidepressant use were 7326, 6405, and 5890, respectively (a total of 19,621 person observations). The analysis on CES-D depressive symptoms (measured only at phases 7 and 9) was based on 4509 participants (or person observations).

We used logistic regression to study the age- and sex-adjusted associations of baseline vascular disease (CHD or stroke) and nonvascular disease with GHQ symptoms at follow-up across each of the three data cycles among participants without GHQ symptoms at the baseline of the cycle. We repeated this analysis with onset of GHQ symptoms before age 65 and at age 65 or later in separate analyses. We also repeated these analyses with onset of CES-D depressive symptoms and use of antidepressants over the follow-up as the outcome measures.

#### Analysis 2: Framingham Risk Scores as Predictors of Depressive Symptoms in Participants Free of Vascular Diseases

Participants were eligible for these analyses if they completed the health questionnaire and attended the screening at any two consecutive phases between phases 3 and 9 (Supplement 1). At the baseline of each of the three screening cycles, we excluded participants who had prevalent depressive symptoms at baseline, those with prevalent CHD or stroke, and those with missing data on risk factors for the Framingham scores at baseline or on depressive symptoms at baseline or follow-up. For the second and third screening cycles, we additionally excluded those with previous depressive symptoms before the baseline of the cycle. In the analysis for GHQ symptoms, there were 4687, 2956, and 3013 participants for the three data cycles (a total of 10,656 person observations). In the analysis of antidepressant use, the corresponding numbers of participants were 6339, 4474, and 4889 (15,702 person observations). The analyses for CES-D depressive symptoms was based on 2786 participants (or person observations).

We constructed Framingham CVD, CHD, and stroke risk scores for each participant and used logistic regression to examine the association of each risk score at baseline with depressive symptoms at follow-up. Crude, age- and sex-adjusted, and multivariably adjusted odds ratios and 95% confidence intervals per 10% absolute increase in risk score were calculated. In the analysis of GHQ symptoms, the sample was large enough to detect, with 90% power at a significance level of .05, an odds ratio of 1.14 for a 10% increase in the Framingham CVD risk score. For depressive symptoms defined by CES-D and antidepressant medication, the corresponding odds ratios were 1.40 and 1.24, respectively.

#### Analysis 3: Subsidiary Analysis

To more comprehensively exclude participants with prevalent or previous depression at baseline, we examined the associations between the Framingham risk scores at phase 5 and CES-D depressive symptoms at phase 7 or phase 9 in a subcohort (*n* = 1635) free of depressive symptoms (i.e., without GHQ symptoms and antidepressant use) and also without a history of depression, based on a psychiatric interview completed at phase 5: the University of Michigan version of the Composite International Diagnostic Interview (33,34). In addition, the effect of a range of baseline characteristics (Supplement 1) on these associations was examined using multivariable adjustments.

## Results

Differences between the analytic sample and the excluded participants were generally small (Table S1 in Supplement 1). Compared with those included, participants excluded from the analyses were more likely to be women, nonwhite, and current smokers, although other characteristics were similar in the two groups. Across the 5-year data cycles, 12.1% of participants without GHQ symptoms developed such symptoms, 4.6% of those without CES-D depressive symptoms had such symptoms at follow-up, and 2.1% of those not on antidepressant treatment started such medication. Of the cases of GHQ symptoms, 5.4% used antidepressant medication, the corresponding proportion being 9.2% for those with CES-D depressive symptoms. Of participants with GHQ symptoms, 47.4% also had CES-D depressive symptoms (Pearson correlation at phase 7 *r* = .64, *p* < .001). Conversely, among participants with CES-D depressive symptoms, 59.9% also had GHQ symptoms.

### Manifest Vascular and Nonvascular Diseases as Predictors of Depressive Symptoms

Table 1 shows results of the association of prevalent manifest vascular and nonvascular diseases with indicators of depressive symptoms. Age- and sex-adjusted odds ratios for new-onset GHQ symptoms, CES-D depressive symptoms, and use of antidepressants were 1.75, 2.02, and 1.50 for participants with prevalent CHD or stroke compared with those without these conditions. The associations of long-term nonvascular disease (excluding comorbid vascular disease) with indicators of subsequent depressive symptoms were similar (range of odds ratios between 1.53 and 1.88).

### Framingham Risk Scores as Predictors of Depressive Symptoms

Table 2 presents age- and sex-adjusted odds ratios for associations of Framingham risk scores with subsequent depressive symptoms in those free of manifest vascular disease. Higher Framingham risk scores were associated with higher odds for new-onset depressive symptoms before age 65 as assessed with CES-D (odds ratios between 1.42 and 3.09, depending on the score). Higher CHD risk score was additionally associated with greater odds of starting antidepressant treatment. In contrast, none of the vascular risk scores were associated with new-onset GHQ symptoms before the age of 65 (odds ratios between 1.03 and 1.17). Furthermore, none of the vascular risk scores had statistically significant associations with GHQ symptoms, CES-D depressive symptoms, or antidepressant medication use after the age of 65 (all *p* values > .35). In unadjusted analyses, no evidence of a significantly greater risk of depressive symptoms before or after the age of 65 among persons with higher vascular risk scores was found (Tables S3 and S4 in Supplement 1). For example, unadjusted odds ratio per 10% increase in the stroke risk score was 1.56 (95% CI .72–3.39) for CES-D depressive symptoms before the age of 65.

### Subsidiary Analysis

After adjustment for multiple covariates (age, sex, marital status, education, socioeconomic status, retirement, cognitive impairment, body mass index, alcohol consumption, menopausal status [women], and nonvascular chronic condition), the association between vascular disease and subsequent CES-D depressive symptoms remained in participants with no prevalent or previous depressive episodes, as indicated by the Composite International Diagnostic Interview (Table 3). Higher stroke risk score was associated with higher odds of onset of CES-D depressive symptoms before (odds ratio 2.30, 95% CI 1.03–5.13), but not after, the age 65. The CVD and CHD risk scores were not associated with subsequent depressive symptoms.

## Discussion

This longitudinal study of British adults shows that manifest cardiovascular disease (CHD and stroke) is associated with increased risk of depressive symptoms. There was also some evidence to suggest an association between higher Framingham stroke risk score and increased onset of depressive symptoms before the age of 65 years. However, the vascular risk scores did not predict later-life depressive symptoms among persons with no manifest vascular disease or history of depression. This finding was based on data from three different vascular risk prediction algorithms—the Framingham CVD, CHD, and stroke scores—and depressive symptoms measured using two survey instruments and information on use of antidepressant medication.

The association between manifest vascular disease and increased risk of depressive symptoms is in agreement with previous studies (6,34–38). The onset of depressive symptoms in those with manifest vascular disease may be the result of vascular pathology, but it is also possible that the diagnosis of a pernicious chronic disease per se and its impact on quality of life induces depressed mood. The second of these explanations is supported in our study by results showing an equally strong and consistent association between long-term nonvascular diseases (excluding vascular comorbidity) and the risk of subsequent depressive symptoms. Indeed, depressive symptoms in the elderly may be a correlate of various disabling conditions (e.g., chronic lung disease, loss of hearing, and loss of vision) (4) in addition to diseases of a vascular origin. Physiological impact of both vascular and nonvascular disease on inflammatory, endocrine, and immune systems may increase vulnerability to depressive symptoms (39,40).

Validated multifactorial risk prediction algorithms, such as the Framingham risk scores, and objective measures of atherosclerosis are used to determine preclinical vascular risk status (41,42). To the best of our knowledge, this is the first large-scale study of a middle-aged population on the association between Framingham risk scores and later-life depressive symptoms. These scores alone did not predict the onset of depressive symptoms at age 65 or older, although the stroke risk score was associated with the onset of depressive symptoms at younger ages. These results were apparent in age- and sex-adjusted and multivariably adjusted models but were weaker in unadjusted models, possibly because greater age and male sex increased the stroke risk score but were related to a lower risk of depressive symptoms in that age range. The association between stroke risk and depressive symptoms warrants further research, as it raises the possibility that cerebrovascular rather than cardiovascular risk status may act as an early marker of depression risk.

A few previous studies have examined the longitudinal association between overall vascular risk and later-life depression using other indicators of vascular risk status. In the Health, Aging and Body Composition study of adults aged 70 or higher, a score combining selected vascular risk factors (body mass index, metabolic syndrome, smoking, and ankle arm index) and vascular diseases predicted higher 2-year incidence of elevated depressive symptoms (7). However, the extent to which this association was attributable to vascular risk factors alone is difficult to assess in such a study design. In the Rotterdam study of older adults followed up for 6 years (11), none of the objective atherosclerosis measures was linked with subsequent depression. This null finding was evident irrespective of whether assessment of depression was based on CES-D or a clinical diagnosis obtained from a psychiatric interview (11). Similarly, in the Leiden 85+ prospective cohort study, ratings of generalized atherosclerosis were not associated with depressive symptoms (43).

In light of this study, as well as data from previous longitudinal studies (11,43), unfavorable preclinical vascular status is not an independent risk factor for later-life depressive symptoms. It is possible that the hypothesized vascular origin of depression in older adults is driven by severe clinical vascular pathology and therefore apparent only in relation to manifest disease or that vascular disease is a correlate rather than cause of depressive symptoms in later life (44–47). Later-life depressive symptoms may also be a heterogeneous multietiological disorder and depression resulting from vascular pathology only a small subset of all later-life depressive disorders in older people. This would lead any association between vascular risk factors and overall later-life depressive symptoms to be diluted, an explanation in keeping with a recent prospective population-based study that showed no significant association between later-life depression and cerebrovascular pathology in postmortem data (48).

### Limitations

There are caveats to the results reported here. We measured depressive symptoms using validated instruments, such as the General Health Questionnaire and CES-D, and information on prescribed antidepressant medication use (26,29,49). These instruments are not designed to make a psychiatric diagnosis of first or recurrent major depression (26,29); they defined only partially overlapping case populations and some misclassification occurred because antidepressants are also prescribed for conditions other than depression. Nevertheless, the associations of manifest CHD and stroke with depressive symptoms in our study are in agreement with previous research linking vascular disease to depressive symptoms and clinical depression (4,35–38,44). Furthermore, in a validation study of 274 elderly participants from our cohort, sensitivity and specificity of the questionnaire measures with depression diagnosed based on structured psychiatric interview as the criterion are high, almost 90% for CES-D depressive symptoms and approximately 80% for GHQ symptoms.

Despite a high response to the survey (range 66% to 88%) at the successive data collection phases, loss to follow-up accumulated over the extended follow-up, as is inevitable in long-term prospective studies. However, differences between the included and excluded participants were generally small. Our study is based on an occupational cohort, which, by its very nature, is healthier than the general population, so the range of vascular risk scores and the range of depressive symptom measurements are likely to be narrower. This being the case, the associations between the Framingham risk scores and depressive symptoms reported here could underestimate the strength of associations in the general population, although similar associations between manifest vascular disease and depressive symptoms in the present dataset and those from the general population suggest that our estimates are likely to be fairly accurate. Due to the relatively low numbers of depressive symptom cases in the present data, we cannot detect weak associations between vascular risk scores and later-life depressive symptoms.

### Clinical Implications

These results suggest that public health measures to improve vascular status will influence the incidence of later-life depression, primarily via reduced rates of manifest vascular disease. Our findings on diagnosed vascular disease support current clinical guidelines to screen patients with coronary heart disease or stroke for depressive symptoms. However, although the stroke risk score was associated with depressive symptoms before the age of 65, we found little evidence to suggest that standard clinical information on preclinical vascular risk status would be helpful in the prediction of depressive symptoms after the age of 65 years. Thus, extending screening of incident later-life depression to include those identified as having a high risk of developing vascular disease was not supported by this study.

## Figures and Tables

**Table 1:** Association of Prevalent Vascular (Coronary Heart Disease or Stroke) and Nonvascular Disease (Long-Standing Illness) with Subsequent Onset of Depressive Symptoms

Predictor	*n*^a^	*n* of Cases	Odds Ratio^b^ (95% CI)	*p* Value
Prevalent CHD/Stroke	Outcome: New-onset GHQ symptoms^c^			
No	12166	1461	1.0	
Yes	664	96	1.75 (1.39, 2.21)	<.001
Long-Standing Illness (Nonvascular)^d^				
No	7095	779	1.0	
Yes	4884	662	1.53 (1.37, 1.72)	<.001
Prevalent CHD/Stroke	Outcome: New-onset CES-D depressive symptoms^e^			
No	4137	253	1.0	
Yes	372	41	2.02 (1.42, 2.98)	<.001
Long-Standing Illness (Nonvascular)^d^				
No	1787	76	1.0	
Yes	2293	177	1.88 (1.41, 2.47)	<.001
Prevalent CHD/Stroke	Outcome: Starting antidepressant treatment^c^			
No	18430	369	1.0	
Yes	1191	34	1.50 (1.04, 2.16)	.03
Long-Standing Illness (Nonvascular)^d^				
No	10088	153	1.0	
Yes	7810	205	1.80 (1.45, 2.23)	<.001

CES-D, Center for Epidemiologic Studies Depression Scale; CHD, coronary heart disease; CI, confidence interval; GHQ, General Health Questionnaire.

a Number of person observations.

b Odds ratios are adjusted for age and sex.

c Across three data cycles (from phase 3 to phase 5; from phase 5 to phase 7; from phase 7 to phase 9).

d Excluding participants with vascular disease (CHD or stroke).

e From phase 7 to phase 9 (one data cycle).

**Table 2:** Association Between Framingham Risk Scores (per 10% Increase) and Subsequent Onset of Depressive Symptoms Before and After Age 65

Outcome	Predictor^a^	Age at Onset <65				Age at Onset ≥65 (Later-Life)			
		*n*^b^	*n* of Cases	Odds Ratio (95% CI)	*p* Value	*n*^b^	*n* of Cases	Odds Ratio (95% CI)	*p* Value
New-Onset GHQ Symptoms	CVD risk score	9445	1184	1.03 (.92, 1.15)	.59	1211	89	.97 (.79, 1.20)	.80
	CHD risk score	9445	1184	1.12 (.99, 1.25)	.06	1211	89	1.03 (.78, 1.36)	.83
	Stroke risk score	9445	1184	1.17 (.84, 1.64)	.35	1211	89	.83 (.54, 1.27)	.38
New-Onset CES-D Depressive Symptoms	CVD risk score	1916	59	1.42 (1.03, 1.96)	.04	870	39	1.09 (.80, 1.47)	.60
	CHD risk score	1916	59	1.44 (1.02, 2.03)	.04	870	39	1.00 (.63, 1.59)	.99
	Stroke risk score	1916	59	3.09 (1.48, 6.47)	.003	870	39	.83 (.43, 1.58)	.57
Starting Antidepressant Treatment	CVD risk score	14,045	283	1.19 (.99, 1.44)	.07	1657	32	1.16 (.85, 1.58)	.35
	CHD risk score	14,045	283	1.24 (1.02, 1.51)	.04	1657	32	1.08 (.68, 1.71)	.75
	Stroke risk score	14,045	283	1.06 (.57, 1.97)	.85	1657	32	.93 (.48, 1.79)	.83

CES-D, Center for Epidemiologic Studies Depression Scale; CHD, coronary heart disease; CI, confidence interval; CVD, cardiovascular disease; GHQ, General Health Questionnaire.

a Odds ratio per each 10% increase in risk score adjusted for age and sex.

b Number of person observations.

**Table 3:** Association of Prevalent Disease and Framingham Risk Scores at Phase 5 with Subsequent Onset of CES-D Depressive Symptoms at Phase 7 or Phase 9 Among Participants with No Current or Previous Depressive Symptoms, No Current or Previous Antidepressant Use, and No History of Depression Based on Psychiatric Interview

Predictor	*n* of Participants	*n* of Cases	Odds Ratio^a^ (95% CI)	*p* Value	Odds Ratio^b^ (95% CI)	*p* Value
	Outcome: New-onset depressive symptoms—all ages					
Prevalent CHD/Stroke						
No	1541	150	1.0		1.0	
Yes	94	15	1.75 (.97, 3.15)	.06	1.82 (1.00, 3.31)	.05
Long-Standing Illness (Nonvascular)^c^						
No	910	78	1.0		1.0	
Yes	598	70	1.41 (1.00, 1.98)	.05	1.40 (.99, 1.99)	.06
	Outcome: New-onset depressive symptoms before age 65					
CVD Risk Score^c^						
Per 10% increase	984	106	1.25 (.90, 1.75)	.19	1.27 (.89, 1.81)	.19
CHD Risk Score^c^						
Per 10% increase	984	106	.96 (.64, 1.46)	.86	.96 (.61, 1.49)	.84
Stroke Risk Score^c^						
Per 10% increase	984	106	2.54 (1.19, 5.43)	.02	2.30 (1.03, 5.13)	.04
	Outcome: New-onset depressive symptoms at age ≥65					
CVD Risk Score^c^						
Per 10% increase	377	28	.86 (.54, 1.37)	.53	1.03 (.65, 1.65)	.89
CHD Risk Score^c^						
Per 10% increase	377	28	.56 (.28, 1.12)	.10	.71 (.35, 1.44)	.33
Stroke Risk Score^c^						
Per 10% increase	377	28	1.37 (.72, 2.60)	.33	1.30 (.62, 2.72)	.37

CES-D, Center for Epidemiologic Studies Depression Scale; CHD, coronary heart disease; CI, confidence interval; CVD, cardiovascular disease.

a Odds ratios are adjusted for age and sex.

b Odds ratios are adjusted for age, sex, marital status, education, socioeconomic status, retirement, cognitive impairment, body mass index, alcohol consumption, and menopausal status for women. Analyses of vascular disease and risk scores as predictors were additionally adjusted for nonvascular chronic condition.

c Excluding participants who have diagnosed vascular disease.
