# Supplementary material for: Vascular Risk Status as a Predictor of Later-Life Depressive Symptoms: A Cohort Study
Source: Biol Psychiatry. 2012 Aug 15;72(4):324–30. doi: 10.1016/j.biopsych.2012.02.005 (PMC3539141; doi:10.1016/j.biopsych.2012.02.005)
Supplement: Supplement 1 [file mmc1.pdf]

Vascular Risk Status as a Predictor of Later-life Depressive Symptoms: A Cohort Study

Supplement

Sample Selection for Analysis 1

Participants were eligible for these analyses if they completed the health questionnaire at any two consecutive phases between Phase 3 and Phase 9 and had no missing data on disease status at the corresponding baseline phases. At each of the 3 data collection cycles, we successively excluded participants who had missing data on General Health Questionnaire (GHQ) symptoms at baseline (*n* = 188, 383 and 102 at phases 3, 5 and 7); those who had GHQ symptoms at baseline (*n* = 1624, 2187 and 2522); or had missing data on GHQ symptoms at follow-up (*n* = 486, 85 and 73). Conversely, participants who did not have missing data or depressive symptoms in one data cycle were eligible to the next data cycle. The analysis was therefore based on 5230, 4029, and 3571 participants in the 1st, 2nd and 3rd data cycles, a total of 12,830 person-observations (Table S1). The corresponding figures for the analysis of antidepressant use were 7326, 6405 and 5890 respectively (a total of 19,621 person-observations). In the analysis on Center for Epidemiologic Studies Depression scale (CES-D) depressive symptoms (measured only at Phases 7 and 9), we excluded prevalent CES-D cases by restricting the analysis to 4509 participants in the last data cycle (4509 person-observations).

Sample Selection for Analysis 2

Participants were eligible for these analyses if they completed the health questionnaire and attended the screening at any two consecutive phases between Phase 3 and Phase 9. At the baseline of each of the 3 screening cycles, we successively excluded participants who had missing data on GHQ symptoms at baseline (*n* = 7, 141 and 66 at phases 3, 5 and 7); those with prevalent coronary heart disease or stroke (*n* = 217, 370 and 518 at Phases 3, 5 and 7, respectively), prevalent or previous GHQ symptoms (*n* = 1485, 1792 and 2163), missing data on risk factors for the Framingham scores at baseline (*n* = 258, 547 and 126); and missing data on GHQ symptoms at follow-up (*n* = 404, 57, and 48), leaving 4687, 2956, and 3013 participants for the analytic samples (a total of 10,656 person-observations) (Table S1). In the analysis of antidepressant use, the corresponding numbers of participants were 6339, 4474 and 4889 after the corresponding exclusions (15,702 person-observations). In the analyses for CES-D depressive symptoms, we excluded prevalent CES-D depressive symptoms by restricting the analysis to the 2786 participants in the last data cycle (a total of 2786 person-observations).

Analysis of Sample Retention and Unadjusted Relationships

Table S2 shows characteristics of the participants included in Analysis 1 (test of prevalence disease-depressive symptoms association) and Analysis 2 (test of risk score-depressive symptoms associations among participants with no vascular disease at baseline) and those excluded from these analyses. Any differences between the groups were relatively small.

Tables S3 and S4 present unadjusted odds ratios for associations of Framingham risk scores and incident depressive symptoms. We found higher scores to be associated with a reduced likelihood of early-onset GHQ symptoms but, as shown in Table S4, these negative associations disappeared after adjustment for age and sex. Irrespective of adjustments, there were no associations between the vascular risk scores and late-life GHQ symptoms. This apparently paradoxical finding is likely to be due to confounding by age, as Framingham risk scores increase with increasing age, corresponding to the higher risk of vascular disease in elderly individuals, but the prevalence of GHQ symptoms decreased between the ages of 40 and 60 and subsequently remained relatively stable at older ages. These contrasting age-related trends explain the unadjusted inverse association of vascular risk with incidence of depression which disappeared after adjustment for age and sex. Unadjusted associations of risk scores with CES-D depressive symptoms and antidepressant use were all null.

Sensitivity Analysis

Sensitivity analysis was based on a subgroup of Phase 5 participants with no current or previous GHQ symptoms, no current or previous use of antidepressant medication and with no history of depression according to the Composite International Diagnostic Interview (CIDI), a total of 1635 participants (1309 men and 326 women at age 45 to 68 years). The following covariates were assessed: marital status (married/co-habiting vs. single vs. widowed/separated/divorced); socioeconomic status (low vs. intermediate vs. high last known occupational position); retirement (yes vs. no); education level (lower secondary school or less vs. higher secondary school vs. university or higher degree); cognitive impairment (the 30-item Mini Mental State Examination < 24); body mass index (weight in kilograms/height in meters^2); alcohol intake (none vs. moderate [1 to 14 units per week in women and 1 to 21 units per week in men] vs. high [>14 units in women and >21 in men]); menopausal status for women (yes vs. no); and non vascular chronic condition (yes vs. no).

Table S1. Study design and number of participants at baseline and follow-up examinations.

Baseline examination	Eligible participants[a]		Number after exclusions[b]		Follow-up examination
Analysis 1 (Vascular & non-vascular diseases as predictors of depressive symptoms)					
Phase 3 (1991-1993)					Phase 5 (1997-1999)
Men	5246	→	3769	→	541 new-onset cases
Women	2282	→	1461	→	289 new-onset cases
Total	7528	→	5230	→	830 new-onset cases
Phase 5 (1997-1999)					Phase 7 (2003-2004)
Men	4699	→	2985	→	338 new-onset cases
Women	1985	→	1044	→	146 new-onset cases
Total	6684	→	4029	→	484 new-onset cases
Phase 7 (2003-2004)					Phase 9 (2008-2009)
Men	4441	→	2661	→	172 new-onset cases
Women	1827	→	910	→	71 new-onset cases
Total	6268	→	3571	→	243 new-onset cases
All Phases – total number of person-observations	20480	→	12830	→	1577 new-onset cases
Analysis 2 (Framingham risk scores as predictors of depressive symptoms)					
Phase 3 (1991-1993)					Phase 5 (1997-1999)
Men	4939	→	3380	→	481 new-onset cases
Women	2119	→	1307	→	253 new-onset cases
Total	7058	→	4687	→	734 new-onset cases
Phase 5 (1997-1999)					Phase 7 (2003-2004)
Men	4170	→	2219	→	255 new-onset cases
Women	1680	→	737	→	94 new-onset cases
Total	5850	→	2956	→	349 new-onset cases
Phase 7 (2003-2004)					Phase 9 (2008-2009)
Men	4230	→	2243	→	133 new-onset cases
Women	1698	→	770	→	57 new-onset cases
Total	5928	→	3013	→	190 new-onset cases
All Phases – total number of person-observations	18836	→	10656	→	1273 new-onset cases

CES-D, Center for Epidemiologic Studies Depression Scale; CHD, coronary heart disease; GHQ, General Health Questionnaire.

[a] Eligible participants are those who attended both the baseline and follow-up examination (see methods section).

[b] Number of participants after exclusion of participants with GHQ-symptoms or missing data (Analysis 1) or with prevalent CHD or stroke (Analysis 2). For analyses of CES-D depressive symptoms and antidepressant medication use, the figures are not exactly the same.

Table S2. Characteristics of the participants in 1991-1993 and the entire Whitehall II population at study inception in 1985-1988.

Characteristic	**Phase 1 characteristics of the cohort**		**Phase 3 characteristics of participants**	
	Excluded from Analysis 2	Included in Analysis 2	Included in Analysis 2	Included in Analysis 1
Number of participants	4545	5763	5230[a]	4687[b]
Mean age (SD), y	44.3 (6.1)	44.6 (6.1)	49.9 (6.1)	49.8 (6.1)
Women, *n* (%)	1790 (39.4)	1623 (28.2)	1461 (27.9)	1307 (27.9)
White, *n* (%)	3853 (87.4)	5120 (91.7)	4658 (91.9)	4171 (91.8)
Mean blood pressure (SD), mm Hg				
Systolic	123.4 (15.3)	122.9 (14.3)	120.8 (13.5)	120.6 (13.5)
Diastolic	77.1 (10.6)	76.8 (9.9)	79.8 (9.3)	79.8 (9.3)
Use of antihypertensive medication, *n* (%)	185 (4.2)	144 (2.6)	326 (6.3)	260 (5.6)
Diabetes mellitus, *n* (%)	57 (1.3)	37 (0.6)	139 (2.9)	121 (2.6)
Current smoker, *n* (%)	1059 (23)	827 (14)	624 (11.9)	552 (11.8)
Total cholesterol, mmol/L	5.98 (1.20)	5.94 (1.14)	6.50 (1.16)	6.49 (1.15)
HDL-cholesterol, mmol/L	1.49 (0.44)[c]	1.49 (0.41)[c]	1.42 (0.41)	1.43 (0.41)
Use of lipid-lowering drugs, *n* (%)	1 (0.0)	1 (0.0)	38 (0.7)	30 (0.6)
Use of antidepressant medication, *n* (%)	-	-	57 (1.1)	46 (1.0)
Atrial fibrillation, *n* (%)	-	-	24 (0.5)	22 (0.5)
Left ventricular hypertrophy, *n* (%)	-	-	212 (4.1)	194 (4.1)
Coronary heart disease (MI or angina), *n* (%)	61 (1.3)	47 (0.8)	132 (2.5)	0 (0.0)
History of stroke, *n* (%)	-	-	8 (0.2)	0 (0.0)
Framingham cardiovascular score, mean (SD)	-	-	-	0.099 (0.074)
Framingham coronary heart disease score, mean (SD)	-	-	-	0.092 (0.065)
Framingham stroke score, mean (SD)	-	-	-	0.034 (0.017)
GHQ-case, *n* (%)	1535 (34)	1209 (21)	0 (0.0)	0 (0.0)
CES-D-depressive symptoms, *n* (%)	-	-	-	-

CES-D, Center for Epidemiologic Studies Depression Scale; GHQ, General Health Questionnaire; HDL, high-density lipoprotein; MI, myocardial infarction.
[a] Of the 5763 participants contributing to analysis 1, 533 participants did not contribute to the first cycle of data from Phase 3 to Phase 5.
[b] Of the 5318 participants contributing to analysis 2, 631 participants did not contribute to the first cycle of data from Phase 3 to Phase 5.
[c] Data available only for a subsample (*n* = 696 excluded from Analysis 2, 1003 included in Analysis 2)).

Table S3. Unadjusted association between Framingham risk scores and onset of depressive symptoms in all age groups.

**Outcome**	**Predictor**[a]	***n***[b]	***n* of cases**	**Odds ratio (95% CI)**	***P*-Value**
New-onset GHQ-symptoms	CVD risk score	10,656	1273	0.69 (0.63, 0.75)	<0.001
	CHD risk score	10,656	1273	0.72 (0.66, 0.80)	<0.001
	Stroke risk score	10,656	1273	0.43 (0.33, 0.55)	<0.001
New-onset CES-D-depressive symptoms	CVD risk score	2786	98	1.06 (0.88, 1.29)	0.52
	CHD risk score	2786	98	1.08 (0.82, 1.41)	0.59
	Stroke risk score	2786	98	1.21 (0.81, 1.81)	0.35
Starting antidepressant treatment	CVD risk score	15,702	315	0.93 (0.81, 1.06)	0.28
	CHD risk score	15,702	315	0.95 (0.81, 1.13)	0.59
	Stroke risk score	15,702	315	0.65 (0.43, 1.00)	0.05

CES-D, Center for Epidemiologic Studies Depression Scale; CHD, coronary heart disease; CI, confidence interval; CVD, cardiovascular disease; GHQ, General Health Questionnaire.

[a] Per 10% increase in risk.

[b] Number of person-observations.

Table S4. Age-stratified analysis: Unadjusted association between Framingham risk scores and onset of depressive symptoms before and after age 65.

		Age at onset <65				Ages ≥65 (Late life)			
**Outcome**	**Predictor**[a]	*n*[b]	*n* of cases	Odds ratio (95% CI)	*P*-Value	*n*	*n* of cases	Odds ratio (95% CI)	*P*-Value
New-onset GHQ-symptoms	CVD risk score	9445	1184	0.67 (0.61, 0.74)	<0.001	1211	89	0.94 (0.78, 1.14)	0.52
	CHD risk score	9445	1184	0.73 (0.65, 0.81)	<0.001	1211	89	0.95 (0.74, 1.22)	0.69
	Stroke risk score	9445	1184	0.38 (0.28, 0.53)	<0.001	1211	89	0.85 (0.56, 1.28)	0.43
New-onset CES-D-depressive symptoms	CVD risk score	1916	59	0.99 (0.72, 1.36)	0.94	870	39	0.99 (0.75, 1.31)	0.94
	CHD risk score	1916	59	1.15 (0.78, 1.69)	0.49	870	39	0.87 (0.57, 1.31)	0.50
	Stroke risk score	1916	59	1.56 (0.72, 3.39)	0.26	870	39	0.82 (0.44, 1.54)	0.53
Starting antidepressant treatment	CVD risk score	14,045	283	0.88 (0.75, 1.04)	0.13	1657	32	1.09 (0.82, 1.45)	0.56
	CHD risk score	14,045	283	0.95 (0.78, 1.16)	0.62	1657	32	0.98 (0.64, 1.48)	0.91
	Stroke risk score	14,045	283	0.42 (0.22, 0.79)	0.008	1657	32	0.96 (0.52, 1.80)	0.91

CES-D, Center for Epidemiologic Studies Depression Scale; CHD, coronary heart disease; CI, confidence interval; CVD, cardiovascular disease; GHQ, General Health Questionnaire.

[a] Per 10% increase in risk.

[b] Number of person-observations.
